# Immediate Effect of Restricted Knee Extension on Ground Reaction Force and Trunk Acceleration during Walking

**DOI:** 10.1155/2021/8833221

**Published:** 2021-07-08

**Authors:** Hiroshi Osaka, Daisuke Fujita, Kenichi Kobara, Tadanobu Suehiro

**Affiliations:** Department of Physical Therapy, Faculty of Rehabilitation, Kawasaki University of Medical Welfare, Kurashiki, Okayama, Japan

## Abstract

Gait parameters calculated from trunk acceleration reflect the features of gait; however, they cannot evaluate the gait pattern corresponding to the gait cycle. This study is aimed at investigating the differences in gait parameters calculated from trunk acceleration during gait corresponding to the gait cycle in healthy subjects with restricted knee extension. Participants included eight healthy volunteers who walked normally (NW) and with knee orthosis that restricted knee extension (ER). The ground reaction force (GRF), joint angles, and trunk acceleration during walking were measured using four force plates, a three-dimensional motion analysis system, and an inertial measurement unit. The peak GRF of the vertical components, joint ranges of motion, and moments of force were analyzed. The root mean square (RMS) and amplitude peak ratio (AR) of autocorrelation function were calculated from the trunk acceleration waveform. The first peak GRF and peak ankle dorsiflexion angles significantly increased during ER. The peak hip extension, knee flexion, knee extension angles, and the peak moment of knee extension significantly decreased during ER compared to that during NW. The acceleration AR significantly decreased during ER compared to that during NW. There was no significant difference in the RMS between the two conditions. The acceleration AR may show the temporal postural structure with restricted knee extension from the terminal stance phase for the ipsilateral limb to the initial stance phase for the contralateral limb. These results suggest that novel metrics for accelerometry gait analysis can reveal gait abnormalities, with restricted knee extension corresponding to the gait cycle.

## 1. Introduction

An inertial measurement unit (IMU) is a small wearable instrument that is used for both gait analysis during rehabilitation [1–3] and activity measurement in daily living [4, 5]. It measures gravitational force and acceleration by combining data from accelerometers, gyroscopes, and magnetometers. IMUs are attached to the body to measure cyclical body movement relative to the center of gravity (COG) by detecting changes in trunk acceleration during walking [6]. Trunk acceleration measurement by IMU during gait represents the characteristics of walking and is useful to identify gait abnormalities [7]. IMUs can be used not only for calculating important gait parameters, such as spatiotemporal parameters [8–10], or for investigating the gait stability and variability between normal and pathological gaits but also for classifying different types of gait patterns, for example, the stroke patient gait pattern [11] or Parkinson's disease gait pattern [12]. These benefits have led to gait analysis with IMUs being widely used in clinical settings and care units. Compared to large-scale systems like three-dimensional motion analysis systems, gait analysis using IMUs has advantages that include a relatively low cost, simplicity of measurement, and the ability to perform the measurement anywhere. These benefits have led to gait analysis with IMUs being widely used in clinical settings and care units.

However, gait analysis using IMUs has several disadvantages. First, the dedicated acceleration analysis software for gait analysis is only an option and the users or researchers need to analyze the obtained acceleration signal data individually. Second, the trunk acceleration used for calculating the walking parameters includes a plurality of walking cycle components that are not easily separated. Therefore, the measured parameters represent the characteristics of the entire walking cycle and they cannot be used to detect an abnormality in a particular part of the gait cycle. The analysis would be much more useful if abnormalities in specific parts of a gait cycle could be identified using an IMU.

Joint contracture in the lower extremity is a major cause of gait abnormality. In older adults, hip and ankle joint movements are reduced during walking leading to reduced peak ankle moments and power generation [13]. Knee osteoarthropathy (OA) is a major disease that causes knee joint contracture, joint stiffness, and decrease in knee extensor muscle strength [14, 15]. Ground reaction force (GRF) during walking and muscle forces affect the external load on the knee [16], increasing mechanical stress on the knee in patients with knee OA. In addition to knee OA flexion contracture, knee adduction moment at the gait stance phase is increased by varus malalignment, resulting in lateral thrust. Furthermore, the pattern of vertical GRF changes in knee OA gait [17]. Specifically, joint contracture might change the ranges of motion of lower extremity joints during walking and also change GRF and moments.

Trunk acceleration during walking shows a similar pattern to the GRF and changes according to the gait cycle. COG displacement correlates with trunk acceleration of the vertical component [6], and COG trajectory can be calculated by analyzing trunk acceleration of this vertical component [18]. Thus, analyzing trunk acceleration of the vertical component may reveal gait abnormalities corresponding to the gait cycle. As far as we know, there have been no studies that have analyzed trunk acceleration corresponding to the gait cycle. If the accelerometer enables gait analysis corresponding to the gait cycle, it may be possible to identify gait abnormalities in more detail.

This study is aimed at investigating differences in the gait parameters calculated from trunk acceleration during gait corresponding to the gait cycle in healthy subjects with restricted knee joints, which simulated the characteristic motion of knee flexion contracture. The authors hypothesized that GRF changes during walking, under knee extension restriction conditions, and that acceleration walking parameters calculated by the novel analysis method can detect the change.

## 2. Methods

### 2.1. Design

#### 2.1.1. Participants

Eight healthy subjects (three males; five females; age, 20.5 ± 0.5 years; height, 161.0 ± 10.8 cm; and body weight, 59.7 ± 11.1 kg) participated in this study. The exclusion criteria were past or present musculoskeletal, neurological, psychological, or cardiopulmonary disease.

Before data collection, all of the procedures were explained to the participants and they signed an informed consent form. This study was approved by Kawasaki University of Medical Welfare Research Ethics Committee (approval number: 17–070). The study was conducted in accordance with the Declaration of Helsinki (1964).

#### 2.1.2. Experimental Procedure

**Figure 1:**
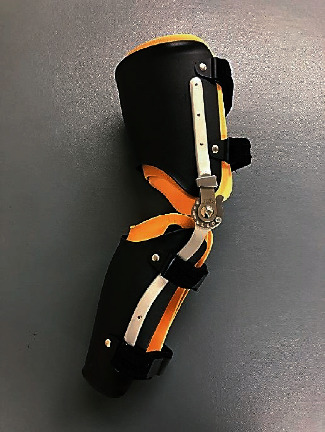
Custom-fabricated knee orthosis. The metal upright knee orthosis can be adjusted by the inner cuff, with the angle restricted by a dial lock joint.

The experiments were performed in a quiet room. All subjects were instructed to walk 10 m. The experimental conditions were normal walking and walking with a custom-fabricated knee orthosis (Figure 1) on the nondominant leg with a 30° restriction to full knee extension (extension restricted (ER)). The nondominant leg for all participants was the left one. The custom-fabricated knee orthosis is a metal upright orthosis that is adjusted by the inner cuff, with the angle restricted by a dial lock joint. GRF is affected by the assigned walking speed [19], which was the same for both the NW and ER conditions for all participants. Prior to measurement, the cadence of the participants walking comfortably was measured. The walking speed for the two conditions was set as a constant by having the participants walk in time with an electronic metronome set to the predetermined cadence.

**Figure 2:**
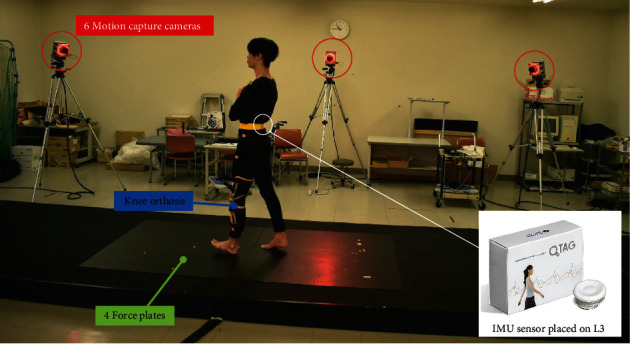
Illustration of synchronized sensor and motion analysis system. IMU: inertial measurement unit.

The GRF, joint angles, and trunk acceleration during walking were measured using four force plates, a three-dimensional (3D) motion analysis system, and IMU, respectively. The synchronized sensor and motion analysis system is illustrated in Figure 2.

Four force plates (MG-1120; ANIMA Co., Tokyo, Japan) were placed at the center of the walkway. The GRF signals were sampled at a rate of 100 Hz.

The joint angles of lower extremities were measured using a 3D motion analysis system (MA8000; ANIMA Co., Tokyo, Japan), and the sampling rate was 100 Hz. Twelve reflective markers were attached to bilateral anatomical locations (acromion process, iliac crest, greater trochanter, lateral knee joint point, lateral malleolus, and 5th metatarsal head).

An IMU including a triaxial accelerometer (Q'z TAG Walk; Sumitomo Electric Industries Ltd., Osaka, Japan) was used to measure trunk acceleration during gait. The IMU was positioned over the third lumbar vertebra (L3) and secured to the subject using an elastic band according to previous studies [2, 4, 9]. The *x*, *y*, and *z*-axes of the accelerometer corresponded to the mediolateral, anteroposterior, and vertical directions, respectively. The acceleration signals were sampled at a rate of 200 Hz and then converted to 100 Hz. The force platform, 3D motion analysis system, and IMU were synchronized, and walking measurement was performed randomly at NW and ER conditions and measured twice for each condition.

#### 2.1.3. Data Analysis

**Figure 3:**
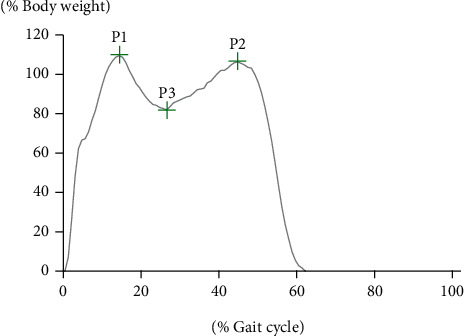
Peak values of vertical ground reaction force. P1: first maximal peak; P2: second maximal peak; P3: minimal peak.

Data was collected for a gait cycle of the nondominant (left) leg during stable gait. The GRF of vertical component peaks, joint ranges of motion, and moments of force were analyzed. The first maximal peak, minimal peak, and second maximal peak values of the GRF of vertical components were calculated (Figure 3). Calculated joint ranges of motion in the sagittal plane were hip flexion, hip extension, knee flexion, knee extension, ankle dorsiflexion, and plantar flexion. Calculated moments of force in the sagittal plane were hip flexion, hip extension, knee flexion, knee extension, and ankle plantar flexion.

In this study, the trunk acceleration waveform was analyzed and two parameters were calculated from the vertical direction component. Acceleration root mean square (RMS) indicates the mean amplitude of the acceleration signal [20]. Therefore, acceleration RMS was calculated as the parameter indicating the amount of postural movement during gait. RMS was derived from equation (1), where *α*^2^(*t*) is the acceleration signal and *T* is the RMS calculation for time duration.

(1) $$\begin{array}{c} \text{RMS}=\sqrt{\frac{1}{T}\int_{t}^{t+T}\alpha^{2}dt}. \end{array}$$

Next, the amplitude ratio (AR) of the auto correlation function (ACF) was calculated as the peak ratio of the acceleration autocorrelation function from the vertical trunk acceleration. ACF was obtained from equation (2), where *x*(*t*) is the mean signal waveform subtracted from the acceleration waveform and divided by the standard deviation, *k* is the delay time, and *n* is the number of observations.

(2) $$\begin{array}{c} \text{ACF}=\frac{1}{n}\overset{n-1}{\underset{t=0}{\sum }}x\left(t\right)x\left(t+k\right). \end{array}$$

**Figure 4:**
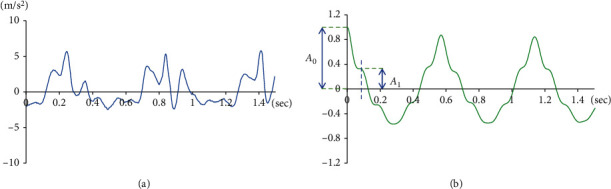
Trunk acceleration and auto correlation function. (a) Trunk acceleration waveform in the vertical direction. (b) Autocorrelation function of trunk acceleration waveform. *A*_0_: the 0th amplitude peak; *A*_1_: first amplitude peak.

Figure 4 shows trunk acceleration and ACF in the vertical direction during walking. The origin amplitude in ACF was defined as the 0th amplitude peak (*A*_0_ in Figure 4), and the apex of the next slope change was defined as the 1st amplitude peak (*A*_1_ in Figure 4). AR was obtained from equation (3) as follows.

(3) $$\begin{array}{c} \text{AR}=\frac{A_{1}}{A_{0}}. \end{array}$$

AR is a novel analysis metric in the present study. Our previous study [21] showed that AR was reduced in subjects with gait abnormalities, such as impairment of balance. The AR value was affected by the first amplitude peak of the autocorrelation function (*A*_1_ in Figure 4), whereas the *A*_1_ value was affected by the amplitude and appearance timing of three characteristic peaks of the acceleration waveform in the vertical direction during walking.

Acceleration RMS and AR were calculated from five strides in the steady state over 10 m of walking. Acceleration signal processing was performed using custom algorithms in MATLAB (version R2017A, MathWorks, Natick, MA, USA).

### 2.2. Statistical Analysis

The Shapiro–Wilk test was used to confirm whether GRF, joint amplitude, and gait parameter calculated from trunk acceleration data approximated a normal distribution. The first peak GRF (P1), the minimal peak (P3), the peak joint angle and moment of hip extension, and the RMS calculated from trunk acceleration did not show a normal distribution. However, other outcomes showed a normal distribution. The paired *t*-test and the Wilcoxon signed-rank test were used to detect differences between NW and ER conditions in GRF, joint amplitude, and gait parameter calculated from trunk acceleration. A *p* value of <0.05 was considered statistically significant. All statistical analyses were performed using IBM SPSS Statistics ver. 23.0 (IBM Corp., Armonk, NY, USA).

## 3. Results

### 3.1. Ground Reaction Force

GRF peak values during walking with and without restricted knee extension by knee orthosis are shown in Table 1. The first peak GRF (P1) was significantly increased in ER compared to NW. There were no significant differences in the minimal peak (P3) and second peak GRF (P2) between the two conditions.

### 3.2. Joint Ranges of Motion

Joint ranges of motion during walking with and without restricted knee extension by knee orthosis are shown in Table 2. The peak ankle dorsiflexion angle was significantly increased in ER compared to NW. The peak angles of hip extension, knee flexion, and knee extension were significantly decreased in ER compared to those in NW.

### 3.3. Joint Moments of Force

Joint moments of force during walking with and without restricted knee extension by knee orthosis are shown in Table 3. The peak moment of knee extension was significantly decreased in ER compared to that in NW. There were no significant differences in peak moments of hip flexion, hip extension, knee flexion, and ankle plantar flexion between the two conditions.

### 3.4. Gait Parameters Calculated from Trunk Acceleration

The gait parameters calculated from trunk acceleration are shown in Table 4. Acceleration AR was significantly decreased in ER compared to that in NW. There was no significant difference in RMS between the two conditions.

## 4. Discussion

This study is aimed at verifying whether gait parameters calculated from trunk acceleration can detect the effects of knee extension restriction on gait in healthy subjects. In this study, kinetic and kinematic parameter variations during walking with or without knee restriction showed significant differences. The first peak of GRF was significantly increased in ER compared to that in NW. GRF of the vertical component represents the changes of COG. The time of the first vertical GRF peak corresponds to the loading response phase in the gait cycle [22]. Here, the knee joint flexes slightly from the near full extension position to accept body weight. The first peak value of GRF is proportional to joint loading during walking and increases with walking speed in knee OA [17]. The loading rate during walking significantly increased by a restriction of 10° of knee flexion [19]. In this study, walking speed between the two conditions had a fixed cadence; the first peak GRF was significantly increased in ER, caused by restricted knee extension at initial contact. Joint amplitude of the lower extremities on the restricted limb decreased compared to that in normal walking. In knee OA, the range of motion and moment force of hip and ankle joints were decreased along with the knee joint [23]. However, the range of motion of ankle dorsiflexion was significantly increased in ER. Participants may have compensated for the knee joint by using the hip and ankle joints to keep the walking speed constant under the two conditions.

In this study, the trunk acceleration waveform during walking was analyzed and two parameters were calculated. Acceleration RMS is a gait parameter widely used in gait analysis that measures trunk acceleration. RMS is the parameter indicating the amount of postural movement during gait [20]. It was found that acceleration RMS can discriminate between stroke patients and healthy individuals [24] and is able to predict fall risk in a nursing home population [25]. In contrast, acceleration AR is a novel analysis metric calculated as the peak amplitude ratio of the acceleration ACF from vertical trunk acceleration.

The autocorrelation coefficient was used as a gait parameter indicating the regularity of walking in previous studies [4, 18]. In this study, the AR was calculated from the autocorrelation function waveform as a novel gait parameter. Our previous study [21] showed that AR was reduced in patients with gait abnormalities, such as impairment of balance. The value of AR was affected by the first amplitude peak of the autocorrelation function (*A*_1_ in Figure 4), and the value of *A*_1_ was affected by the amplitude and appearance timing of three characteristic peaks of the acceleration waveform in the vertical direction during walking. Trunk acceleration during walking shows a similar pattern to the composite GRF waveform in the vertical component. The three characteristic peaks of the acceleration waveform during walking (Figure 4) are composed of the first peak (P1 in Figure 3) and the second peak (P2 in Figure 3) of GRF in the vertical component. The first (P1) and second (P2) vertical GRF peaks correspond to the loading response phase and terminal stance phase, respectively [22]. The three characteristic peaks of the acceleration waveform during walking appear between the terminal stance in the ipsilateral limb and loading response in the contralateral limb during the gait cycle. Therefore, this parameter might indicate the smoothness of weight transfer from the ipsilateral to contralateral limb during walking. In this study, acceleration RMS was not significantly different between the two conditions; nevertheless, AR was significantly decreased in the ER condition. Acceleration RMS indicates the average amplitude of the acceleration waveform signal calculated from a set interval, for example, the entire gait cycle. Therefore, RMS is the amount of postural movement of the entire walk. With knee ER, hip extension was significantly decreased and the hip flexion angle was increased. In spite of trunk fluctuations in the sagittal plane increasing with knee restriction, RMS could not detect any gait abnormalities.

In our results, the first peak GRF was significantly different between the two conditions. GRF and trunk acceleration in vertical components are correlated during walking, and AR reflected changes in GRF and was able to detect changes in gait associated with unilateral knee extension restriction more sensitively than RMS.

This study has several limitations. We only recruited healthy participants and did not analyze subjects with knee flexion contracture. Further research is needed for subjects with knee flexion contractures. Acceleration AR is a novel metric for gait analysis; therefore, further measurement and subject analyses with various gait abnormalities are necessary and the validity of the findings must be verified.

## 5. Conclusions

This study suggests that novel metrics for accelerometry gait analysis can detect gait abnormalities corresponding to the gait cycle. Acceleration AR reflected changes in GRF and was able to detect changes in gait more sensitively than conventional metrics. The method described in our study may be useful for assessing the benefits of exercise therapy and rehabilitation.

## Figures and Tables

**Table 1:** GRF peak (% body weight) during walking.

Variable	NW	ER	*p* value	Effect size	Power
P1	99.5 ± 15.2	107.4 ± 5.6	0.025^∗^	0.60	0.45
P2	103.2 ± 16.0	107.5 ± 5.9	0.503	0.39	0.26
P3	74.2 ± 10.4	78.1 ± 2.7	0.674	0.25	0.16

Values are presented as mean ± standard deviation. NW: normal walking; ER: extension restricted; P1: first maximal peak; P2: second maximal peak; P3: minimal peak; GRF: ground reaction force; ^∗^*p* < 0.05.

**Table 2:** Peak joint ranges of motion during walking.

Variable (degrees)	NW	ER	*p* value	Effect size	Power
Hip flexion	20.3 ± 7.3	25.3 ± 4.5	0.067	0.77	0.62
Hip extension	6.4 ± 5.9	1.9 ± 3.9	0.036^∗^	0.92	0.75
Knee flexion	61.7 ± 4.5	55.8 ± 8.4	0.049^∗^	0.84	0.69
Knee extension	−1.3 ± 5.2	−11.4 ± 2.9	0.004^∗∗^	1.49	0.98
Ankle DF	10.7 ± 5.2	16.3 ± 7.5	0.007^∗∗^	1.32	0.96
Ankle PF	38.7 ± 6.1	35.7 ± 7.6	0.251	0.44	0.30

Values are presented as mean ± standard deviation. NW: normal walking; ER: extension restricted; DF: dorsiflexion; PF: plantar flexion; ^∗^*p* < 0.05; ^∗∗^*p* < 0.01.

**Table 3:** Peak joint moments of force during walking.

Variable (Nm/kg)	NW	ER	*p* value	Effect size	Power
Hip flexion	0.37 ± 0.10	0.40 ± 0.14	0.174	0.53	0.39
Hip extension	0.85 ± 0.14	0.85 ± 0.15	0.575	0.00	0.05
Knee flexion	0.34 ± 0.13	0.52 ± 0.17	0.057	0.80	0.66
Knee extension	0.38 ± 0.12	0.15 ± 0.14	<0.001^∗∗^	2.54	1.00
Ankle PF	1.49 ± 0.19	1.45 ± 0.19	0.057	0.81	0.66

Values are presented as mean ± standard deviation. NW: normal walking; ER: extension restricted; PF: plantar flexion; ^∗∗^*p* < 0.01.

**Table 4:** Gait parameters calculated from trunk acceleration.

Variable	NW	ER	*p* value	Effect size	Power
RMS	2.42 ± 0.48	2.44 ± 0.38	0.889	0.05	0.07
AR	0.39 ± 0.19	0.28 ± 0.14	0.020^∗^	1.06	0.85

Values are presented as mean ± standard deviation. NW: normal walking; ER: extension restricted; RMS: root mean square; AR: amplitude ratio of auto correlation function; ^∗^*p* < 0.05.

## Data Availability

The data used to support the findings of this study are included within the article. Results of the conceptual review are available upon request from the corresponding author.
